# Phytochemicals as alternatives to antibiotics against major pathogens involved in bovine respiratory disease(BRD)and bovine mastitis(BM)

**DOI:** 10.6026/97320630015032

**Published:** 2019-02-03

**Authors:** Karthic Rajamanickam, Jian Yang, Meena Kishore Sakharkar

**Affiliations:** 1 Drug Discovery and Development Research Group, College of Pharmacy and Nutrition, University of Saskatchewan, 107 Wiggins Road, Saskatoon, SK S7N 5E5, Canada

**Keywords:** Bovine respiratory disease, bovine mastitis, bacterial pathogen, phytochemical, MIC

## Abstract

Bovine respiratory disease (BRD) and bovine mastitis (BM) are the most common and costly infectious diseases in beef cattle and dairy cattle, respectively. In the current study, we evaluated the antimicrobial activity of seven phytochemicals against twelve BRD- and/or BMcausing bacterial pathogens. Our results show that allyl isothiocyanate, benzyl isothiocynate, cinnamaldehyde and eugenol are effective against most of the BRD- and/or BM-causing bacterial pathogens and could be repurposed as alternatives to antibiotics for the prevention/elimination of BRD and BM in feedlots.

## Background

Bovine respiratory disease (BRD) and bovine mastitis (BM) are the most common and costly infectious diseases in beef cattle and dairy cattle, respectively [1,2]. The major bacterial pathogensof BRD are Mannheimia haemolytica, Pasteurella multocida and Haemophilus somni; whereas the major bacterial pathogensof BM are Mycoplasma bovis, Staphylococcus aureus, Streptococcus uberis and Escherichia coli [3,4]. A metaphylactic injection of antibiotics upon animal arrival is widely used to prevent BRD or BM [5]. However, with increased consumer concern on antibiotic usage in beef and dairy products, Health Canada has introduced a new regulation that a veterinary prescription is required to purchase any livestock antibiotic from December 2018 (Beef Cattle Research Council, 2018) [6]. Therefore, it is urgent to identify alternatives to antibiotic for the prevention of BRD and BM in feedlots. Phytochemicals, which are secondary metabolites in plants, are emerging as a valuable resource in finding antibiotic alternatives as they are relatively safe and do not leave residues. In this study, we evaluated the antimicrobial activity of seven phytochemicals against twelve BRD- and/or BM-causing bacterial strains, including two clinical isolates of Mycoplasma bovis [7].

## Methodology

### Materials

Gallic acid was purchased from ThermoFisher Scientific (Ottawa, ON, Canada). Allyl isothiocyanate, benzyl isothiocyanate, cinnamaldehyde, eugenol, quercetin and tannic acid were purchased from Sigma-Aldrich Canada (Oakville, ON, Canada). Mannheimia haemolytica ATCC 29702, Pasteurella multocida ATCC 43137, Escherichia coli ATCC 25422, Staphylococcus aureus ATCC 29213, Staphylococcus epidermidis ATCC 12228, Streptococcus dysgalactiae ATCC 43078, Streptococcus uberis ATCC 19436, Enterococcus faecium ATCC 700221, Enterococcus faecalis ATCC 29212 and Pseudomonas aeruginosa ATCC 27853 were purchased from Cedarlane Canada (Burlington, ON, Canada). Mycoplasma bovis (clinical isolate 137.2) and Mycoplasma bovis (clinical isolate147.3) were kindly provided by Dr. Murray Jelinski (University of Saskatchewan).

### Determination of minimum inhibitory concentration (MIC)

The MICs of the phytochemicals against the BRD- and BM-causing bacteria were determined using standard broth micro-dilution assay as outlined by the Clinical and Laboratory Standards Institute (CLSI). Mycoplasma bovis strains were cultured in PPLO broth; whereas the other bacterial strains were cultured in Brain Heart Infusion broth (BHIB). All bacteria strains were sub-cultured at 37°C overnight and then OD_565_ of the bacterial suspensions was adjusted to 0.5 McFarland turbidity with the culture media (approximate cell density: 1.5x10^8^ CFU/mL) using normal saline as a control. For each bacterial strain, 100 µLculture media was added to each well of a 96-well plate with subsequent addition of 5 µL/well of the adjusted bacterial suspension. Then, the bacterial samples were treated with the phytochemicals with concentration ranging from 3.9 µg/mL to 1000 µg/mL. Untreated bacterial samples were used as a negative control. The culture plates were incubated at 37°C for 18-24 h (Mycoplasma bovis: 48-72 h) before OD_655_ was taken for each well using a Bio-Rad iMark Microplate Reader (Bio-Rad Laboratories, Inc., Mississauga, ON, Canada). The readings were double-checked using a SensititreVizion Digital MIC Viewing System (Thermo Fisher Scientific, Ottawa, ON, Canada).

## Results and discussion:

The global concern on antibiotic usage in beef and dairy industry has led to many countries to ban/limit the use of antibiotics as growth promoters (European Commission, 2005; Beef Cattle Research Council, 2018)[6,8]. Various substances, including phytochemicals and herbal plants, have been proposed as potential alternatives of antibiotics. In the current study, we evaluated the antimicrobial activity of seven phytochemicals, allyl isothiocyanate, benzyl isothiocyanate, cinnamaldehyde, eugenol, quercetin and tannic acid, against twelve BRD- and/or BM-causing bacterial strains. As shown in Table 1, benzyl isothiocyanate, cinnamaldehyde and eugenol show the broadest spectrums against the bacterial pathogens, followed by allyl isothiocyanate. Benzyl isothiocyanate was active against all bacterial strains except S. aureus. Benzyl isothiocyanate has MIC ranging from 15.6 µg/mL for P. multocida to 1000 µg/mL for E. coli and P. aeruginosa. Cinnamaldehyde was active against all bacterial strains, with MIC ranging from 62.5 µg/mL for P. multicida to 500 µg/mL for E. coli, S. aureus, E. faecium, E. faecalis and P. aeruginosa. Eugenol was also effective against all bacterial pathogens except P. aeruginosa, however, the activity was much weaker compared to benzyl isothiocyanate and cinnamaldehyde. Allyl isothiocynate was effective against M. haemolytica, P. multocida, M. bovis, S. epidermidis, S. dysgalactiae and S. uberis with MIC ranging from 31.3 µg/mL for P. multocida to 1000 µg/mL towards S. uberis. As previously reported [9-11], the antimicrobial mechanism is also likely to be affecting membrane permeability for allyl isothiocynate and disrupting of energy metabolism for benzyl isothiocynate, cinnamaldehyde and eugenol against the BRD- and/or BM-causing bacterial pathogens. The antimicrobial activity of gallic acid, quercetin and tannic acid exhibited the least spectrums against the bacterial pathogens. Gallic acid was only active against M. haemolytica and M. bovis (clinical isolate 147.3) with MIC at 500 µg/mL and 250 µg/mL, respectively. Quercetin was only active against P. multocida with MIC at 12.5 µg/mL. Tannic was active towards M. haemolytica (MIC: 500 µg/mL) and the two clinical isolates of M. bovis (MIC: 250 µg/mL). In summary, the current study shows that phytochemicals, especially benzyl isothiocyanate and cinnamaldehyde, could be repurposed as alternatives of antibiotics in preventing/eliminating BRD and BM.

## Conclusion

In conclusion, our experimental results indicate that allyl isothiocyanate, benzyl isothiocyanate, cinnamaldehyde and eugenol are effective against several clinical pathogens involved in bovine mastitis and bovine respiratory diseases in dairy farms. Here, it is important to mention that these phytochemicals have also been reported to have anti-biofilm activity. Furthermore, they can be further explored as combination drugs for currently used antibiotics for BM/BRD.

## Disclosure statement:

The authors declare that no conflict of interest exists.

## Funding Source:

Agriculture Development Fund (ADF) grant from the Province of Saskatchewan, Canada, and Accelerate Internships from Mitacs Canada and SaskMilk (Regina, SK, Canada).

## Ethical Approval:

Not applicable.

## Figures and Tables

**Table 1:** The MIC (µg/mL) values of seven phytochemicals, allyl isothiocyanate, benzyl isothiocyanate, cinnamaldehyde, eugenol, quercetin and tannic acid, against the BRD- and/or BM-causing bacterial pathogens.

S. No	Name of the bacterium				Minimum Inhibitory Concentration (µg/mL)			
		Allyl Isothiocyanate	Benzyl Isothiocyanate	Cinnamaldehyde	Eugenol	Gallic acid	Quercetin	Tannic acid
1	Mannheimia haemolytica ATCC 29702	125	62.5	125	250	500	-	500
2	Pasteurella multocida ATCC 43137	31.3	15.6	62.5	250	-	12.5	-
3	Mycoplasma bovis (Clinical Isolate 137.2)	500	125	250	500	-	-	250
4	Mycoplasma bovis (Clinical Isolate 147.3)	125	125	125	500	250	-	250
5	Escherichia coli ATCC 25422	-	1000	500	500	-	-	-
6	Staphylococcus aureus ATCC 29213	-	-	500	1000	-	-	-
7	Staphylococcus epidermidis ATCC 12228	500	250	500	1000	-	-	-
8	Streptococcus dysgalactiae ATCC 43078	125	62.5	125	500	-	-	-
9	Streptococcus uberis ATCC 19436	1000	250	250	1000	-	-	-
10	Enterococcus faecium ATCC 700221	-	500	500	1000	-	-	-
11	Enterococcus faecalis ATCC 29212	-	500	500	1000	-	-	-
12	Pseudomonas aeruginosa ATCC 27853	-	1000	500	-	-	-	-
